# Supplementary material for: Characteristics of Diaphragmatic and Chest Wall Motion in People with Normal Pulmonary Function: A Study with Free-Breathing Dynamic MRI
Source: J Clin Med. 2022 Dec 8;11(24):7276. doi: 10.3390/jcm11247276 (PMC9785439; doi:10.3390/jcm11247276)
Supplement: Supplementary file 1 [file jcm-11-07276-s001.zip › Supplementary.pdf]

Supplementary Video S1. Visualization of of dynamic MRI in quiet breathing on the coronal plane in a 50-year-old non-smoker.

Supplementary Video S2. Visualization of of dynamic MRI in deep breathing on the coronal plane in a 50-year-old non-smoker.

Supplementary Video S3. Visualization of of dynamic MRI in quiet breathing on the sagittal plane in a 50-year-old non-smoker.

Supplementary Video S4. Visualization of of dynamic MRI in deep breathing on the sagittal plane in a 50-year-old non-smoker.
